# Evaluation of Safety for Scanning Carbon-Ion Radiotherapy in Hemodialysis Patients With Prostate Cancer

**DOI:** 10.7759/cureus.22214

**Published:** 2022-02-14

**Authors:** Yosuke Takakusagi, Makito Suga, Yohsuke Kusano, Kio Kano, Satoshi Shima, Keisuke Tsuchida, Nobutaka Mizoguchi, Itsuko Serizawa, Daisaku Yoshida, Tadashi Kamada, Shinichi Minohara, Hiroyuki Katoh

**Affiliations:** 1 Department of Radiation Oncology, Kanagawa Cancer Center, Yokohama, JPN; 2 Division of Radiation Therapy Technology, Kanagawa Cancer Center, Yokohama, JPN; 3 Section of Medical Physics and Engineering, Kanagawa Cancer Center, Yokohama, JPN

**Keywords:** in-room ct, prostate cancer, hemodialysis, carbon-ion radiotherapy, safety

## Abstract

Background/Aim

The efficacy and safety of carbon-ion radiotherapy (CIRT) for prostate cancer have already been demonstrated. The number of hemodialysis (HD) patients is increasing. Although the toxicity of CIRT in HD patients may be more severe, it has been insufficiently investigated. Therefore, we retrospectively analyzed the safety of CIRT for HD patients with prostate cancer in the present study.

Materials and methods

Five HD patients with prostate cancer who underwent CIRT at the Kanagawa Cancer Center during November 2015-2020 were included in this study. CIRT was delivered by the raster scanning method (sCIRT). Adverse events were assessed using the Common Terminology Criteria for Adverse Events version 5.0. The dose-volume histogram (DVH) parameters of the target volume and normal organs were evaluated between initial planning computed tomography (CT) and in-room CT images.

Results

In the acute phase, Grade 1 genitourinary toxicity was recorded in one patient. In the late phase, Grade 1 genitourinary toxicity was recorded in two patients. No gastrointestinal toxicities were noted during the follow-up period. In-room CT analysis revealed no significant differences among all DVH parameters of the target volume and normal organs when compared with the treatment plan dose.

Conclusions

The safety of sCIRT for prostate cancer in HD patients was investigated in the present study. In-room CT analysis suggested the robustness of the treatment plan. According to the present results, sCIRT for prostate cancer can be safely performed in HD patients.

## Introduction

Radiotherapy (RT) is one of the definitive treatments for prostate cancer [1]. The advancement in treatment techniques has affected an increase in the dose to the target volume without increasing the dose to the surrounding normal organs [2]. Especially, carbon-ion RT (CIRT) involves a sharp dose distribution, which is represented by the Bragg peak, and a high biological effect relative to that in with conventional X-rays [3-5]. Owing to these characteristics of CIRT, CIRT has been expected to possess higher therapeutic efficacy and safety for prostate cancer. In fact, several past reports have demonstrated the usefulness of CIRT for prostate cancer [6-9].

The number of dialysis patients due to chronic kidney disease has been increasing across the world, exceeding 1 million count in the year 2010 [10]. In Japan, the number of dialysis patients has also been increasing, with a report of >340,000 patients in 2018 [11]. Approximately 98% of these patients were on hemodialysis (HD) [11]. As reported earlier, the incidence of cancer is relatively higher in HD patients than in the general population [12,13]. Patients with HD tend to suffer from multiple comorbidities in addition to renal disease, and their general conditions tend to be poor. Therefore, the treatment options are limited for HD patients with cancer. RT is expected to be a promising treatment option for HD patients with cancer. However, there are limited reports on the safety of RT for HD patients [14]. Moreover, the safety of CIRT in HD patients remains to be ascertained. Because HD patients have no or very poor urination, their bladder volume is small. As a result, CIRT for prostate cancer in HD patients may cause severe genitourinary (GU) toxicity due to irradiation of a large portion of the bladder [15]. In addition, several studies have demonstrated that a small bladder volume requires a higher dose to the intestine in pelvic RT [16,17]. Thus, gastrointestinal (GI) toxicity is a concern in CIRT for prostate cancer in HD patients. Therefore, in the present study, we retrospectively investigated the toxicity in HD patients treated with CIRT for prostate cancer at our institution. We also evaluated the dose-volume histogram (DVH) to the target volume and normal organs by using images obtained using computed tomography (CT) installed in the treatment room (in-room CT) during the treatment period of CIRT. A preprint of this article was previously posted to the Research Square server on December 29, 2021 (https://www.researchsquare.com/article/rs-1153921/v1).

## Materials and methods

Patients

The present study was approved by the institutional review board of the Kanagawa Cancer Center (approval number: 2021-85). HD patients with prostate cancer who were treated with CIRT at the Kanagawa Cancer Center during December 2015-2020 were enrolled in the present study. The relevant clinical records were collected in October 2021. The eligibility criteria for this study were as follows: (i) HD performed at the time of CIRT initiation, (ii) histological diagnosis of prostate adenocarcinoma, (iii) cT1bN0M0 to T3bN0M0 according to the seventh UICC classification, and (iv) no previous treatment for prostate cancer excluding androgen deprivation therapy (ADT). Patients with peritoneal dialysis were excluded from the study. The D'Amico classification was applied to determine the risk groups [18].

CIRT

The detailed method of CIRT is as described in our previous study [9]. Briefly, the clinical target volume (CTV) was defined as the entire prostate and proximal seminal vesicle, while the planning target volume (PTV) was expanded 5 mm superoinferior and posterior to the CTV and 10 mm anterior and lateral to the CTV. The total dose was set to 51.6 Gy (relative biological effectiveness; RBE) in 12 fractions and the PTV was covered by ≥95% of the prescribed dose. CIRT was delivered by the raster scanning method (sCIRT). Carbon-ion beams were delivered from both the right and left sides of the patient. The bladder, rectum, and intestine were identified as organs at risk (OARs). The rectum was defined as the rectum that existed in the area where the PTV was enlarged by 1 cm in the superoinferior direction. The small intestine and sigmoid colon together were defined as the intestine. The dose constraint for the rectum was aimed at the volume irradiated with 80% of the prescribed dose (V80%) <10 cc, while, for intestine, the maximum dose (Dmax) was <70% dose of the prescribed dose. In cases where the PTV and intestine were extremely close, the PTV was modified to meet the dose constraints of the intestine. Patients without urinary function were not subjected to any pre-treatment restrictions on drinking or urination for the treatment. Patients with preserved urinary function were instructed not to urinate before treatment as much as possible. No bladder infusion was performed. The rectum was emptied as much as possible using a laxative and an anti-flatulent before each session, and enema was performed if the patient did not defecate within 24 h of the treatment.

In-room CT was performed at the first, fifth, and ninth treatment sessions. The acquired in-room CT images were transferred to the MIM maestro software version 7.0 (MIM Software Inc., Cleveland, OH, USA), and rigid image fusion was performed by bone matching with the initial planning CT. On each in-room CT image, an experienced radiation oncologist delineated the CTV, bladder, rectum, and intestine. Dose distribution was accordingly calculated on the in-room CT images without changing the irradiation conditions as determined in the initial treatment planning. The calculation results were confirmed by two or more radiation oncologists. The dose of each structure was evaluated using DVH. The dose covering 98% of the target volume (D98%), D95%, D50%, D2%, and the homogeneity index (HI) were estimated for CTV. HI was calculated using the following formula: D2 − D98/D50 [19]. The percentage of the structure volume receiving at least 10 Gy (RBE) [V10Gy (RBE)], V20Gy (RBE), V30Gy (RBE), V40Gy (RBE), V50Gy (RBE), and the mean dose (Dmean) for the bladder and rectum as well as the Dmax for the intestine were estimated as the DVH parameters for OARs. V80% for the rectum was also calculated. DVH parameters were compared between the treatment planning CT and in-room CT.

ADT

Patients with high-risk prostate cancer were treated by urologists with ADT, which consisted of a combined androgen blockade (CAB) with anti-androgen therapy and medical castration with such luteinizing hormone-releasing hormone agonists. The high-risk group patients received neoadjuvant ADT for 4-8 months, followed by adjuvant ADT for a total of two years.

Follow-up

Urologists and radiation oncologists followed up the patients every three months after the completion of sCIRT. Prostate-specific antigen (PSA) was measured at each follow-up. According to the Phoenix definition, PSA recurrence was defined as the nadir PSA level plus 2 ng/mL [20]. The observation period and the time to the events were calculated since the initiation of sCIRT. Toxicity was assessed using the Common Terminology Criteria for Adverse Events version 5.0. Toxicity <3 months after the initiation of sCIRT was defined as acute toxicity, while toxicity after three months was defined as late toxicity. The worst grade of toxicity was evaluated as the final grade of toxicity.

Statistical analysis

The DVH parameters between the initial plan and in-room CT were compared using the Mann-Whitney U-test. p <0.05 was considered to indicate statistical significance. Statistical analysis was performed using the STATA software (version 13.1, College Station, TX, USA).

## Results

Patient characteristics

Five patients were enrolled in this study. The patient characteristics are summarized in Table 1.

**Table 1:** Patient characteristics PSA: prostate specific antigen; GS: Gleason score; HD hemodialysis; ADT: androgen deprivation therapy; CAB: combined androgen blockade

No.	Age	iPSA (ng/mL)	T stage	GS	D'Amico classification	HD duration (year)	Urination (mL/day)	ADT	Follow- up (months)
1	71	19.2	3a	4 + 4	High	22	-	CAB	24
2	66	46.3	2a	3 + 4	High	33	-	CAB	22
3	68	6.581	1c	4 + 5	High	11	-	CAB	21
4	75	20.561	2c	4 + 4	High	30	<150	CAB	15
5	64	19.66	2c	5 + 4	High	6	<500	CAB	12

The median age of the subjects was 68 (range: 66-75) years. All patients were classified as high-risk groups according to the D'Amico classification parameters. The median duration of HD at the initiation of sCIRT was 22 (range: 6-33) years. The recorded primary diseases included diabetic nephropathy, polycystic kidney disease, chronic glomerulonephritis, and congenital renal atrophy in two, one, one, and one patient, respectively. Two of the five patients recorded a residual urinary function. ADT was performed in all patients with neoadjuvant and adjuvant CAB. The median follow-up duration was 21 (range: 12-24) months. No death or PSA recurrence was recorded during the observation period.

Toxicity

The cases of toxicities are summarized in Table 2.

**Table 2:** Toxicity GU: genitourinary; GI: gastrointestinal

	Acute toxicity, grade			Late toxicity, grade		
No.	GU	GI	Other	GU	GI	Other
1	0	0	0	0	0	0
2	0	0	0	0	0	0
3	1	0	0	1	0	0
4	0	0	0	1	0	0
5	0	0	0	0	0	0

Grade 1 GU toxicity was recorded as acute toxicity (patient no. 3). No other acute toxicity was noted. Two cases of Grade 1 GU were noted as late toxicity (patients no. 3 and 4). Patient no. 3 had acute urethral pain, which continued for three months after sCIRT, and hence judged as late toxicity. This patient’s toxicity resolved spontaneously at the next visit. Patient no. 4 complained of gross hematuria at five months after sCIRT. The patient did not visit any hospital at the onset of hematuria, and no urinalysis or cystoscopy was performed. He experienced only one episode of gross hematuria, which subsequently disappeared without any treatment. No Grade 2 or higher toxicity was recorded in either acute or late phase. No specific toxicity due to ADT was observed during the observation period.

In-room CT analysis

In-room CT was performed only before dialysis in patient no. 3 and only after dialysis in patient no. 4. In other patients, in-room CT was performed before or after dialysis. The DVH parameters of CTV in the initial plan CT and in-room CT are summarized in Table 3.

**Table 3:** DVH parameters for CTV in plan CT and in-room CT DVH: dose-volume histogram; CT: computed tomography; CTV: clinical target volume; SD: standard deviation; RBE: relative biological effectiveness

DVH parameters	Plan CT (mean ± SD)	In-room CT (mean ± SD)	p-Value
CTV			
D98_%_ [Gy (RBE)]	50.8 ± 0.4	50.0 ± 1.5	0.458
D95_%_ [Gy (RBE)]	51.1 ± 0.3	50.9 ± 0.6	0.760
D50_%_ [Gy (RBE)]	51.6 ± 0.0	51.6 ± 0.0	0.315
D2_%_ [Gy (RBE)]	52.0 ± 0.1	52.0 ± 0.1	0.513
Homogenity index	0.02 ± 0.0	0.04 ± 0.03	0.275

The DVH parameters of CTV were not significantly different between the two groups. The DVH parameters of OARs in the initial plan CT and in-room CT are summarized in Table 4.

**Table 4:** DVH parameters for normal organs in plan CT and in-room CT DVH: dose-volume histogram; CT: computed tomography; SD: standard deviation; RBE: relative biological effectiveness

DVH parameters	Plan CT (mean ± SD)	In-room CT (mean ± SD)	p-Value
Bladder			
V10_Gy (RBE)_ (%)	67.7 ± 26.9	60.0 ± 19.4	0.150
V20_Gy (RBE)_ (%)	60.6 ± 25.7	50.8 ± 17.9	0.206
V30_Gy (RBE)_ (%)	52.5 ± 22.9	42.7 ± 16.4	0.206
V40_Gy (RBE)_ (%)	42.3 ± 18.5	33.9 ± 139	0.239
V50_Gy (RBE)_ (%)	24.3 ± 10.6	18.4 ± 8.2	0.150
Dmean [Gy (RBE)]	28.0 ± 11.4	23.8 ± 8.1	0.150
Rectum			
V10_Gy (RBE)_ (%)	26.1 ± 6.6	27.4 ± 9.2	0.695
V20_Gy (RBE)_ (%)	17.7 ± 4.4	19.1 ± 8.2	0.571
V30_Gy (RBE)_ (%)	11.2 ± 2.6	12.9 ± 7.4	0.965
V40_Gy (RBE)_ (%)	5.4 ± 1.1	7.6 ± 6.3	0.827
V50_Gy (RBE)_ (%)	0.0 ± 0.0	2.5 ± 3.3	0.755
Dmean [Gy (RBE)]	8.4 ± 2.0	9.2 ± 3.7	0.458
V80_%_ (cc)	2.2 ± 0.4	3.8 ± 3.7	0.827
Intestine			
Dmax [Gy (RBE)]	15.6 ± 13.5	13.6 ± 16.1	0.695

**Figure 1:**
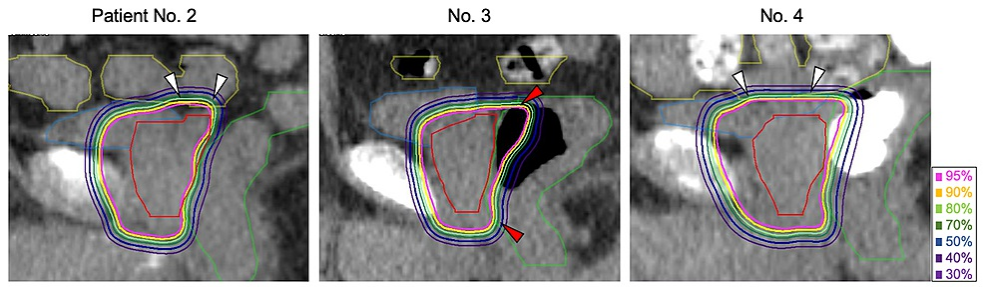
Dose distribution in cases wherein the dose constraint exceeded. In-room CT sagittal images of three patients are shown. Patients no. 2 and 4 received high doses to the nearby intestine (yellow line, white arrows). In patient no. 3, the rectum (green line) enlarged because the rectal gas was irradiated with a high dose (red arrows).

The DVH parameters of all OARs were not significantly different between the two groups. There were three cases in which the dose distribution in the in-room CT did not meet the dose constraints. The dose distributions of these three cases are indicated in Figure 1.

All these cases were recorded only once in different patients. In patient no. 3, the V80% of the rectum exceeded the constraint of 10 cc because the rectal volume was enlarged as a result of gas filling in the rectum; the V80% was 2.6 cc in the treatment plan, but 11.8 cc in the in-room CT. In patients no. 2 and 4, the Dmax of the intestine exceeded the constraint of 36.12 Gy (RBE) because the intestine was closer to the target volume than that in planning CT. The Dmax of the intestine in patients no. 2 and 4 was 20.6 Gy (RBE) and 27.2 Gy (RBE) in the initial treatment plan and 49.4 Gy (RBE) and 42.1 Gy (RBE) in the in-room CT, respectively. In all but these three cases, the doses for the rectum and intestine met the constraints.

## Discussion

In this study, we investigated the safety of sCIRT for prostate cancer in HD patients. No severe toxicity was observed. The analysis of dose distribution using in-room CT demonstrated comparable dose distribution to the initial plan. These results suggested that sCIRT for prostate cancer was safely performed in the HD patients. To the best of our knowledge, there have been no reports on the safety of CIRT in HD patients; the present study is the first report of its kind.

The dialysis tubing, dialysis membranes, and other equipment used for HD patients can cause inflammation [20]. In addition, several factors including cytokines from uremia can cause chronic inflammation [21]. Such inflammation has been reported to increase the cardiovascular risk in HD patients [22]. The acute toxicity of RT is related to inflammation. Moreover, tissue damage based on vascular injury is one of the causes of late toxicity from RT. It has also been reported that patients with diabetes, which is the most common underlying cause of HD, have higher toxicity after RT for prostate cancer [23]. Therefore, both acute and late toxicities may occur more frequently in HD patients treated with RT.

There have been a very few studies on RT for HD patients; for instance, a past study on RT for various primary sites in 56 HD patients revealed that both acute and late toxicities were clinically acceptable [14]. However, severe Grade 5 infections were recorded in four of these 56 patients. In this past study, patients who received chemotherapy were also included. Infections accounted for >20% of all deaths among HD patients [11]. In addition to the immune system abnormalities inherent in HD patients, treatment-induced leukopenia was presumed to be the cause of these severe infections. There have been several case reports on chemoradiation therapy (CRT) for HD patients. In a report on CRT with low-dose cisplatin for recurrent cervical cancer, no adverse events were observed during a six-month follow-up period [24]. In a case report of lung cancer treated with CRT using oral etoposide, adverse events during the treatment period were found to be within acceptable limits; however, post-treatment follow-up was not performed and late toxicity was unknown [25]. A case report on the long-term outcomes of >4 years after CRT for esophageal cancer revealed a good local control and a few adverse events [26]. In the present study, no serious adverse events were recorded after sCIRT. This may be due to the reduction of excessive dose to normal tissues by the favorable dose distribution of CIRT. These reports may suggest that adverse events of RT are acceptable even in HD patients. However, past reports on the clinical outcomes after RT in HD patients are scarce, warranting further studies.

The risk of GU and GI toxicity due to low bladder volume and reduced rectal reproducibility owing to unstable voiding are concerns in RT for prostate cancer in HD patients. In a study on intensity-modulated RT (IMRT) for prostate cancer, an association between V70Gy and GU toxicity has been demonstrated [15]. On the other hand, in another study, empty bladder was associated with non-inferiority in GU toxicity after RT when compared to full bladder [27]. Another study reported that the hotspots in the bladder served as a risk factor for GU toxicity [28]. Although the association between bladder irradiated volume and GU toxicity remains controversial, GU toxicity may require attention if high doses are administered to a large area of the bladder. In addition, RT in the empty bladder can induce increased intestinal doses. In a study of RT for prostate cancer using the four-field box technique, a higher frequency of higher dose in the intestine was recorded during irradiation with an empty bladder rather than with a full bladder [16]. A study of IMRT for cervical cancer suggested that the volume irradiated with 45 Gy in an empty bladder was larger than that in a full bladder [17]. In the present study, the analysis of dose distribution of in-room CT revealed cases wherein the intestine dose exceeded the dose constraint. Although no GI toxicity was observed clinically in the present study, careful observations are warranted for the possibility of unexpectedly high intestine doses. Moreover, in patient no. 3, the increased volume due to gas accumulation in the rectum increased the rectal V80%. Patients with HD were found to be prone to defecation disorders such as constipation and diarrhea [29]. The enlargement of the rectal volume due to constipation and gas retention may increase the rectal dose more than expected.

There are several limitations to this study, including the small number of patients, the short observation period, inclusion of a single center, and the retrospective nature of the study. In addition, based on a study of the robustness of prostate cancer from our institution, a total of three in-room CTs were performed during sCIRT [30]. Therefore, the dose distribution in each treatment session could not be accurately evaluated. The safety of CIRT in HD patients remains unclear, requiring further investigation.

## Conclusions

Our results validated that sCIRT for prostate cancer was safely performed in the HD patients. The DVH analysis using in-room CT generally reproduced the initial treatment plan. However, attention should be paid to the possibility of unexpectedly high doses in the intestine and rectum in such cases.
